# Muzzling or Rabies

**Published:** 1897-12-18

**Authors:** 


### The Hospital

A Journal of

The Medical Sciences and Hospital Administration.

Vol. XXIII.—No. 586.] Edited by Sir Henry Burdett, K.C.B., and Solomon C. Smith, M.D., M.R.C.P. [Dec. 18, 1897.

### Muzzling or Rabies.

Notwithstanding Mr. Long's recent and very clear account of the grounds on which the muzzling order now in operation was promulgated, and of the benefits which it may be expected to produce, the agitation for its abandonment has not wholly subsided, and even seems to have been increased by circumstances which, to ordinary minds, afford fresh proof, if any were needed, of its necessity. But the "Anti" is always with us, and the latest evidence of its existence is furnished by an advertisement in the "agony" column of the *Times* paper, which for downright foolishness is scarcely to be matched, even by any other production of the school from which it emanates. It runs as follows:—

"The muzzle has been proved a failure by the recent cases of rabies in London. That a Board of Agriculture should legislate for the dogs of a city is a flagrant absurdity. The County Council elections are approaching. Electors who wish the right to legislate for local dogs (which is a municipal and not an agricultural matter) restored to the Council are requested to write to," &c., &c.

The recent cases of rabies in London, so far from having proved the muzzle a failure, have occurred in consequence of an unmuzzled rabid dog having escaped from control, and having bitten at least five other dogs and some human beings, so that, in all probability, the full amount of mischief permitted by its unmuzzled state has not yet had time to declare itself. How or where it became rabid has not been made known; but in all probability it came from some part of England in which, on account of the absence of rabies for a long period, the order, unwisely as most people believe, has not yet been enforced. And the obvious objection to the County Council as a legislative authority, even for "local dogs" (what is a "local" dog?), and even if the electors could confer the desired power upon the elected, is that the latter could not possibly exert authority beyond the limits of their ordinary jurisdiction, and hence that the general or national extinction of rabies would be even less probable than it is under the partial exemptions from muzzling which are the weak points in the present arrangements of the Government. A central authority is plainly necessary, even for the proper selection of exempted districts, if such there ought to be; and it would be difficult to show why the Board of Agriculture is less adapted than any other department for the performance of the duty which has been assigned to it. The advertiser has, probably, only just discovered that dogs do not grow in fields, like turnips or mangel-wurzel, and has been stimulated by this newly-acquired knowledge to attempt bringing it into practical application. Dogs, even "local" ones, he probably argues, are not agricultural products, and therefore they cannot reasonably be dealt with by the Board of Agriculture. He forgets that the Board is the department of Government which has at its disposal the services of the most distinguished veterinary surgeons of the country, and which, in all matters relating to the health of animals, is guided by their advice; while the President, like every other member of the Ministry of similar rank, is almost certain to be a gentleman with a full knowledge of rural affairs, habitually keeping and using dogs for purposes of pleasure and of sport, and naturally, therefore, in complete sympathy with dog owners as a body. Mr. Long has expressed this sympathy over and over again; and has declared, in many of his speeches, that nothing but the clearest evidence, and the prospect of a great national benefit, would induce him to support the orders to which so much objection has been made. In our own opinion, these orders err on the side of over-indulgence. We do not believe that the country will ever be free from rabies until far more stringent measures have been adopted. The danger arises mainly from stray curs of uncertain ownership, and from dogs which, although they might be claimed by somebody, are not properly cared for by their proprietors. We should like to see the dog tax increased in amount, with due indulgence to persons who require dogs for purposes of trade or occupation, and with the issue of receipts in the form of collar badges, the colour of which might be varied every year, and which would enable a policeman to see at a glance whether the tax for any particular dog had been paid. If it had not the dog should be summarily destroyed. By the strict enforcement of such a system we should obtain a state of things analogous to that which exists in Norway, where not only is rabies unknown, but where the dogs seen in the streets are handsome, well-bred, and well-cared for animals, not sufficiently numerous to be the nuisances they often are in London. The Norwegians adopt very stringent precautions with regard to dogs imported into their territory, insisting upon ample certification of health, and also, we believe, upon a period of quarantine; and, even in that most democratic country, we have never heard that the control of canine hygiene is vested in local authorities, which would be liable, in this matter as in many others, to oscillate between unreasoning indulgence and unreasoning panic. The average county councillor has no more love for hydrophobia than his neighbours; and, if a scare were once started in his district, even "local" dogs would be likely to receive only scant mercy at his hands.

